# Triglyceride–Glucose Index and Ischemic Stroke Burden in Permanent Versus Paroxysmal Atrial Fibrillation: A Real-World Retrospective Cohort Study

**DOI:** 10.3390/metabo16070482

**Published:** 2026-07-09

**Authors:** Horia Silviu Branea, Ciprian Ilie Rosca, Daniel Florin Lighezan, Violeta Ariana Nicoras, Adrian Sebastian Zus, Romina Georgiana Bita, Daniel-Dumitru Nisulescu

**Affiliations:** 1 Department of Internal Medicine I—Medical Semiotics II, “Victor Babeș” University of Medicine and Pharmacy, Eftimie Murgu Sq. No. 2, 300041 Timișoara, Romania; 2 Department V, Internal Medicine I—Discipline of Medical Semiology I, Center of Advanced Research in Cardiology and Hemostaseology, “Victor Babeș” University of Medicine and Pharmacy, Eftimie Murgu Sq. No. 2, 300041 Timișoara, Romania; 3 Doctoral School Medicine–Pharmacy, “Victor Babeș” University of Medicine and Pharmacy, Eftimie Murgu Sq. No. 2, 300041 Timișoara, Romania; ariana.nicoras@umft.ro (V.A.N.); daniel.nisulescu@umft.ro (D.-D.N.); 4 Department of Histology, Faculty of Medicine, Vasile Goldiș Western University of Arad, 310025 Arad, Romania; 5 Cardiology Department, “Victor Babeș” University of Medicine and Pharmacy, 300041 Timișoara, Romania; 6 2nd Department of Dentistry, “Victor Babeș” University of Medicine and Pharmacy, 300041 Timișoara, Romania

**Keywords:** atrial fibrillation, permanent atrial fibrillation, paroxysmal atrial fibrillation, triglyceride–glucose index, TyG index, ischemic stroke, recurrent stroke, cerebrovascular burden, insulin resistance, cardiometabolic heterogeneity, population dependence

## Abstract

**Highlights:**

**What are the main findings?**

- Permanent atrial fibrillation was associated with a substantially higher ischemic stroke burden than paroxysmal atrial fibrillation. The TyG index was higher in paroxysmal AF but was not independently associated with any ischemic stroke or recurrent ischemic stroke.

**What are the implications of the main findings?**

- Stroke burden in heterogeneous AF cohorts may be driven more by AF phenotype and accumulated clinical risk than by the TyG index alone. The prognostic utility of TyG may be population-dependent and should be tested in selected cardiometabolic subgroups.

**Abstract:**

**Background:** Atrial fibrillation (AF) is a major substrate for ischemic stroke, but cerebrovascular risk is heterogeneous and influenced by AF phenotype, comorbidities, anticoagulant exposure, renal function, and metabolic risk. The triglyceride–glucose index (TyG index) is a simple surrogate marker of insulin resistance calculated from fasting triglycerides and fasting plasma glucose. Whether the TyG index is associated with ischemic stroke burden and recurrent cerebrovascular events in patients with different AF phenotypes remains insufficiently characterized. **Objective:** This study aimed to evaluate the association between the TyG index and ischemic stroke burden in patients with paroxysmal and permanent AF, with particular focus on recurrent ischemic stroke. **Methods:** We performed a retrospective observational analysis of patients with AF divided according to AF phenotype: paroxysmal AF and permanent AF. Clinical, biological, therapeutic, and cerebrovascular variables were extracted from an existing real-world clinical database. The TyG index was calculated as ln [fasting triglycerides (mg/dL) × fasting plasma glucose (mg/dL)/2]. Triglycerides and fasting plasma glucose were measured during hospitalization; therefore, the analysis was framed as a retrospective cross-sectional association with recorded ischemic stroke burden, not as a prospective prediction study. The main outcome was any ischemic stroke. Secondary outcomes included recurrent ischemic stroke, defined as ≥2 recorded ischemic stroke events, and stroke burden categorized as no stroke, single stroke, or recurrent stroke. Continuous variables were reported as mean ± standard deviation and compared using independent samples *t*-tests or one-way analysis of variance, as appropriate. **Results:** After patient-level de-duplication, 1031 unique patients with AF were identified: 310 with paroxysmal AF and 721 with permanent AF. The strict TyG-eligible analytical cohort included 941 patients: 295 with paroxysmal AF and 646 with permanent AF. Permanent AF was associated with a higher ischemic stroke burden than paroxysmal AF. Any ischemic stroke was recorded in 325/646 patients with permanent AF compared with 79/295 patients with paroxysmal AF (50.3% vs. 26.8%, *p* < 0.001). Recurrent ischemic stroke was also more frequent in permanent AF (7.7% vs. 4.1%, *p* = 0.035). Among stroke-positive patients, the proportion with recurrent stroke was similar between permanent and paroxysmal AF (15.4% vs. 15.2%, *p* = 0.966). The TyG index was significantly higher in paroxysmal AF than in permanent AF (8.93 ± 0.69 vs. 8.73 ± 0.61, *p* < 0.001), but it did not differ significantly across stroke burden categories. In logistic regression, the TyG index was not associated with any ischemic stroke or recurrent ischemic stroke in unadjusted, age/sex-adjusted, AF phenotype-adjusted, or clinically adjusted models. **Conclusions:** In this real-world retrospective cohort, permanent AF was associated with a substantially higher ischemic stroke burden than paroxysmal AF. However, the TyG index was not independently associated with ischemic stroke occurrence or recurrence. Although patients with paroxysmal AF had a more adverse lipid metabolic profile and higher TyG index values, this did not translate into a higher TyG-related cerebrovascular burden. These findings suggest that the TyG index alone has limited utility as a marker of ischemic stroke burden in heterogeneous AF cohorts and that its prognostic value may depend on cardiometabolic phenotype, adiposity distribution, AF substrate, anticoagulant exposure, and the clinical context in which it is applied.

## 1. Introduction

Atrial fibrillation (AF) is one of the most clinically important arrhythmias encountered in cardiovascular practice and represents a major substrate for ischemic stroke, systemic embolism, heart failure progression, hospitalization, cognitive decline, and mortality. Contemporary AF management is no longer restricted to rhythm or rate control, but is increasingly framed as an integrated strategy addressing comorbidities and risk factors, prevention of stroke and thromboembolism, symptom control, and longitudinal reassessment. The 2024 ESC Guidelines for the management of atrial fibrillation place comorbidity and risk factor control at the beginning of the AF-CARE pathway, followed by prevention of stroke and thromboembolism and individualized rhythm or rate control strategies [1].

The relationship between AF and ischemic stroke is clinically complex. Although cardioembolism from the left atrium and left atrial appendage is the classical mechanism, cerebrovascular risk in AF is not determined by the arrhythmia alone. Age, hypertension, diabetes mellitus, heart failure, renal dysfunction, vascular disease, prior cerebrovascular disease, left atrial structural remodeling, and anticoagulant exposure all contribute to the observed risk. This is why current guidelines recommend that decisions regarding oral anticoagulation should be based primarily on the patient’s thromboembolic risk profile rather than on AF pattern alone [1,2]. Nevertheless, AF phenotype remains clinically relevant, because permanent or non-paroxysmal AF is often associated with higher arrhythmic burden, more advanced atrial disease, and greater comorbidity load than paroxysmal AF.

Previous studies have suggested that AF burden and AF pattern may influence thromboembolic risk. Patients with non-paroxysmal AF have generally shown higher rates of stroke and mortality than those with paroxysmal AF in clinical trial and registry analyses, although this relationship is partly mediated by differences in age, comorbidities, and structural cardiovascular disease [3]. In patients with paroxysmal AF, higher measured AF burden has also been associated with increased thromboembolic risk, independent of established clinical scores [4]. These data support the concept that AF phenotype and AF burden may provide additional clinical context, but they do not replace conventional risk stratification or the need for individualized anticoagulant therapy.

In parallel, metabolic dysfunction has emerged as an important contributor to both AF development and vascular complications. Insulin resistance, dyslipidemia, visceral adiposity, chronic low-grade inflammation, oxidative stress, endothelial dysfunction, platelet activation, and prothrombotic remodeling may contribute to atrial remodeling and cerebrovascular disease. The TyG index, calculated from fasting triglycerides and fasting plasma glucose, has been proposed as a simple and inexpensive surrogate marker of insulin resistance [5,6]. It is particularly attractive for retrospective and real-world datasets because triglycerides and glucose are routinely measured, whereas fasting insulin and direct insulin resistance measurements are rarely available in clinical practice.

Recent literature has associated the TyG index with several cardiovascular and cerebrovascular outcomes. A systematic review and meta-analysis reported that higher TyG index values were associated with increased ischemic stroke risk in the general population and with higher risk of stroke recurrence and mortality among patients with ischemic stroke [7]. In AF-specific literature, meta-analytic data have also suggested an association between higher TyG index values and AF risk, although heterogeneity between studies is substantial and routine clinical use remains insufficiently established [8]. These findings provide a rationale for evaluating whether TyG index may help characterize cerebrovascular risk among patients with AF.

However, the incremental value of TyG index in established AF populations remains uncertain. Stroke in AF is multifactorial, and the metabolic contribution to cerebrovascular burden may be attenuated or confounded by age, AF phenotype, renal function, heart failure, anticoagulant therapy, and prior vascular disease. Moreover, most available studies have evaluated incident AF, incident ischemic stroke, or prognosis after acute ischemic stroke, whereas fewer data address the relationship between TyG index and ischemic stroke burden in cohorts stratified by AF phenotype.

The present study therefore evaluates the association between the TyG index and ischemic stroke burden in a real-world retrospective cohort of patients with paroxysmal and permanent AF. The study focuses not only on the presence of ischemic stroke, but also on recurrent ischemic stroke, defined as two or more recorded ischemic stroke events. Because waist circumference, body weight, and height were not available in the database, the conicity index and composite TyG–conicity index could not be calculated. Accordingly, the present analysis focuses on the TyG index as an accessible metabolic marker and examines whether it is associated with ischemic stroke occurrence or recurrence beyond the differences attributable to AF phenotype and clinical risk profile.

## 2. Materials and Methods

### 2.1. Study Design and Source Database

This was a retrospective observational study based on a secondary analysis of an existing real-world clinical database of patients with atrial fibrillation (AF). The source database consisted of a single-center registry generated at the Municipal Emergency Hospital in Timisoara, Romania, and included patients admitted to the medical ward with a diagnosis of AF between 1 January 2015 and 31 December 2016. Before the initiation of the original registry, approvals from the hospital management and the Ethics Committee of the “Victor Babes” University of Medicine and Pharmacy, Timisoara, were obtained.

For the present analysis, the database was re-queried to evaluate the association between the TyG index and ischemic stroke burden across AF phenotypes. Triglycerides and fasting plasma glucose were measured during hospitalization; therefore, in stroke-positive patients, TyG was measured after the recorded stroke event or during the same clinical episode. Accordingly, the study was interpreted as a retrospective cross-sectional association analysis rather than as a prospective prediction study. Prediction terminology was avoided because the temporal sequence between triglyceride/glucose measurements and ischemic stroke occurrence could not be fully established for all patients.

### 2.2. Study Population and Cohort Construction

The principal inclusion criterion was the presence of AF recorded in the admission file. There were no restrictions regarding sex, age, ethnicity, socioeconomic status, previous medication, or comorbidities. Patients admitted without an AF diagnosis were not eligible. The initial registry included 1111 AF-related records; after data verification, exclusion of incomplete records, and patient-level de-duplication of repeated admissions using AF phenotype and patient number, 1031 unique patients with AF were retained for the present analysis.

Patients were eligible for the TyG-specific analytical cohort if they had: (1) documented AF phenotype; (2) available fasting triglyceride and fasting plasma glucose values allowing calculation of the TyG index; (3) available information regarding ischemic stroke events; and (4) sufficient clinical data for baseline characterization. Patients were excluded from TyG-specific analyses if triglyceride or glucose values were missing, non-numeric, or biologically implausible. The strict TyG-eligible analytical cohort included 941 patients, of whom 295 had paroxysmal AF and 646 had permanent AF.

To assess potential selection bias related to missing triglyceride or fasting plasma glucose measurements, patients included in the TyG-eligible cohort were compared with patients excluded from TyG-specific analyses. This comparison included demographic variables, AF phenotype, major comorbidities, renal function, cerebrovascular history, and antithrombotic/lipid-lowering therapy, where available. The results are reported in Supplementary Table S1.

### 2.3. Atrial Fibrillation Diagnosis and Phenotype

AF was diagnosed using 12-lead electrocardiography at admission. Patients were classified according to the predefined group variable in the database. Lot 1 corresponded to paroxysmal AF, while Lot 2 corresponded to permanent AF. The present study therefore compares two clinically distinct AF phenotypes rather than incident versus prevalent AF.

### 2.4. Clinical, Biological, and Therapeutic Variables

Demographic, clinical, biological, and therapeutic variables were extracted from the existing database. The variables considered for descriptive and adjusted analyses included age, sex, residence, hypertension, overweight status, obesity categories, diabetes mellitus, dyslipidemia, renal function estimated using eGFR, oral anticoagulant therapy, antiplatelet therapy, statin therapy, antihypertensive treatment classes, NYHA class III–IV heart failure, coronary artery disease, carotid stenosis, CHA_2_DS_2_-VASc score, HAS-BLED score, ATRIA score, total cholesterol, HDL-C, LDL-C, triglycerides, fasting plasma glucose, and the TyG index. Continuous body mass index and waist circumference values were not systematically available in the database; therefore, adiposity was analyzed using the available recorded categorical variables for overweight and obesity. Target blood pressure achievement could not be reliably assessed because standardized outpatient or pre-admission blood pressure values and individualized blood pressure targets were not systematically recorded.

Oral anticoagulant exposure was recorded, but time in therapeutic range for vitamin K antagonist users could not be calculated because serial INR values were not systematically available. The TyG index was interpreted as treated metabolic status during hospitalization, since lipid-lowering therapy may influence triglycerides and antidiabetic therapy may influence fasting plasma glucose.

The presence of ischemic stroke and related cerebrovascular history was recorded if the diagnosis had been documented in current admission files or previous medical evaluation records by a neurologist, psychiatrist, radiologist, or other responsible specialist, according to the retrospective validation framework used for the source database. Renal function was evaluated using eGFR. Where chronic kidney disease staging was required, CKD-EPI/KDIGO-based definitions were applied.

### 2.5. TyG Index Calculation

TyG index was calculated using the standard formula [5,6]:

TyG index = ln [fasting triglycerides (mg/dL) × fasting plasma glucose (mg/dL)/2]

Only patients with available triglyceride and fasting plasma glucose values were included in TyG-specific analyses. The TyG index was analyzed as a continuous variable and, for logistic regression, per 1-standard deviation increase. In this cohort, the standard deviation of TyG index was 0.64.

Because waist circumference, body weight, and height were not available in the database, the conicity index, TyG-BMI, TyG–waist circumference, and the TyG–conicity index could not be calculated. This limitation is clinically relevant because adiposity distribution and composite metabolic–anthropometric indices may capture cardiometabolic risk more accurately than the TyG index alone [9].

### 2.6. Cerebrovascular Outcomes

The primary outcome was the presence of any ischemic stroke, defined using recorded ischemic stroke event variables in the database. Secondary outcomes included recurrent ischemic stroke, defined as at least two recorded ischemic stroke events, and ischemic stroke burden categorized as no ischemic stroke, single ischemic stroke, or recurrent ischemic stroke.

Ischemic stroke diagnosis was based on clinical documentation and cranial CT confirmation according to routine European diagnostic criteria. However, detailed etiological stroke mechanism classification, such as cardioembolic, large artery, small vessel, mixed, or undetermined stroke, was not systematically available.

Because the registry was retrospective, ischemic stroke was interpreted as cerebrovascular burden associated with the clinical profile of the patient rather than as an incident event prospectively predicted by baseline TyG index. This distinction is important because the TyG index can be influenced by nutritional status, acute illness, treatment, and metabolic control at the time of measurement.

### 2.7. Covariates and Circularity Considerations

The following covariates were considered in adjusted analyses, depending on completeness and event count: age, sex, AF phenotype, hypertension, diabetes mellitus, dyslipidemia, NYHA class III–IV heart failure, oral anticoagulant therapy, and eGFR. The CHA_2_DS_2_-VASc score was not included in the primary logistic regression models where ischemic stroke was the dependent variable because previous stroke or transient ischemic attack is a component of the score. Including this score in such models would introduce partial circularity and overadjustment. To address the baseline imbalance between AF phenotypes, an additional extended sensitivity model was constructed. This model incorporated the available adiposity and hypertension-related variables from the database. Because continuous BMI, waist circumference, and target blood pressure achievement were not available, recorded overweight/obesity status, hypertension grade variables, and the number of antihypertensive drug classes were used as the available proxies for adiposity burden and hypertension treatment profile. Hypertension grade was used instead of adding multiple highly collinear hypertension indicators.

### 2.8. Statistical Analysis

Continuous variables were reported as mean ± standard deviation. Categorical variables were reported as counts and percentages. Between-group comparisons were performed using independent samples *t*-tests for continuous variables and chi-square or Fisher’s exact test for categorical variables, as appropriate. For comparisons across three stroke burden categories, one-way analysis of variance was used. This analytical approach was consistent with the descriptive and regression-based framework used for the database-derived analyses.

Binary logistic regression was used for dichotomous outcomes: any ischemic stroke versus no ischemic stroke and recurrent ischemic stroke versus no recurrent ischemic stroke. The TyG index was analyzed per 1-standard deviation increase. Planned regression models were: Model 1, unadjusted TyG index; Model 2, the TyG index adjusted for age and sex; Model 3, the TyG index adjusted for age, sex, and AF phenotype; and Model 4, clinical adjustment including age, sex, AF phenotype, hypertension, diabetes mellitus, dyslipidemia, NYHA III–IV heart failure, oral anticoagulant therapy, and eGFR. Missing data were handled using a complete-case approach for each analysis. No imputation was performed because the study was based on a retrospective registry and the missingness mechanism could not be reliably assumed to be random. For logistic regression models, model convergence was verified, and multicollinearity among covariates was assessed using variance inflation factors. The number of outcome events per model was also reviewed, particularly for recurrent ischemic stroke, where the smaller event count required cautious interpretation of fully adjusted estimates. The data is reported in Supplementary Table S2.

A two-sided *p*-value <0.05 was considered statistically significant. Given the exploratory, retrospective, and database-derived nature of the study, the results were interpreted with emphasis on effect sizes, confidence intervals, and internal consistency across models rather than isolated *p*-values.

## 3. Results

### 3.1. Study Population

After patient-level de-duplication, the study cohort included 1031 unique patients with AF, of whom 310 had paroxysmal AF and 721 had permanent AF. The strict TyG-eligible analytical cohort included 941 patients with available triglyceride and fasting plasma glucose values, allowing TyG index calculation. Among these, 295 patients had paroxysmal AF and 646 patients had permanent AF.

In the TyG-eligible cohort, any ischemic stroke was recorded in 404 patients, while recurrent ischemic stroke, defined as at least two recorded ischemic stroke events, was identified in 62 patients. The subsequent analyses were performed in this TyG-eligible cohort.

### 3.2. Baseline Clinical Characteristics According to AF Phenotype

Baseline characteristics according to AF phenotype are presented in Table 1. Patients with permanent AF were significantly older than those with paroxysmal AF, with a mean age of 73.6 ± 10.0 years versus 69.7 ± 10.8 years, respectively (*p* < 0.001). Sex distribution was not significantly different between groups, although female patients were numerically more frequent in the permanent AF group.

Permanent AF was associated with a higher global thromboembolic and bleeding risk profile. CHA_2_DS_2_-VASc score, HAS-BLED score, and ATRIA score were all significantly higher in the permanent AF group (all *p* < 0.001). Renal function was also less favorable in permanent AF, with lower eGFR values compared with paroxysmal AF: 53.2 ± 21.9 versus 57.7 ± 22.4 mL/min/1.73 m^2^ (*p* = 0.004).

Regarding comorbidities, hypertension was highly prevalent in both groups and showed borderline statistical significance, being numerically more frequent in paroxysmal AF (89.5% versus 84.8%; *p* = 0.054). Diabetes mellitus and dyslipidemia were significantly more frequent among patients with paroxysmal AF. Conversely, NYHA class III–IV heart failure was more frequent in permanent AF: 45.8% versus 26.8% (*p* < 0.001).

Therapeutic profiles also differed between AF phenotypes. Oral anticoagulant therapy was more frequent in permanent AF than in paroxysmal AF (88.2% versus 77.3%; *p* < 0.001). In contrast, statin therapy, aspirin therapy, and clopidogrel therapy were all more frequent among patients with paroxysmal AF.

Additional adiposity, hypertension grade, and antihypertensive treatment variables are presented in Supplementary Table S3. Recorded overweight or obesity was present in 100/295 patients with paroxysmal AF and 225/646 patients with permanent AF (33.9% vs. 34.8%, *p* = 0.838). The distribution of hypertension grade did not differ substantially between groups. Diuretic therapy was more frequent in permanent AF, whereas angiotensin receptor blocker therapy was more frequent in paroxysmal AF. Continuous BMI, waist circumference, and target blood pressure achievement could not be reported because these variables were not systematically available in the source database.

### 3.3. Biological and Metabolic Profile According to AF Phenotype

The biological and metabolic profile is shown in Table 2. Patients with paroxysmal AF had significantly higher total cholesterol, LDL-C, triglyceride values, and TyG index values than patients with permanent AF. Total cholesterol was 160.4 ± 52.2 mg/dL in paroxysmal AF versus 149.7 ± 48.4 mg/dL in permanent AF (*p* = 0.003). LDL-C was also higher in paroxysmal AF: 99.1 ± 43.5 versus 91.0 ± 38.8 mg/dL (*p* = 0.007).

Triglyceride values were significantly higher in the paroxysmal AF group, with a mean of 127.5 ± 77.9 mg/dL compared with 105.8 ± 58.7 mg/dL in permanent AF (*p* < 0.001). Fasting plasma glucose showed no statistically significant difference between groups: 146.2 ± 71.9 mg/dL in paroxysmal AF versus 139.3 ± 64.8 mg/dL in permanent AF (*p* = 0.162). Consequently, TyG index was significantly higher in paroxysmal AF than in permanent AF: 8.93 ± 0.69 versus 8.73 ± 0.61 (*p* < 0.001).

These findings indicate that patients with paroxysmal AF had a more adverse lipid metabolic profile, while patients with permanent AF had a more advanced clinical risk phenotype characterized by older age, higher risk scores, worse renal function, and more frequent advanced heart failure.

### 3.4. Cerebrovascular Burden According to AF Phenotype

**Figure 1:**
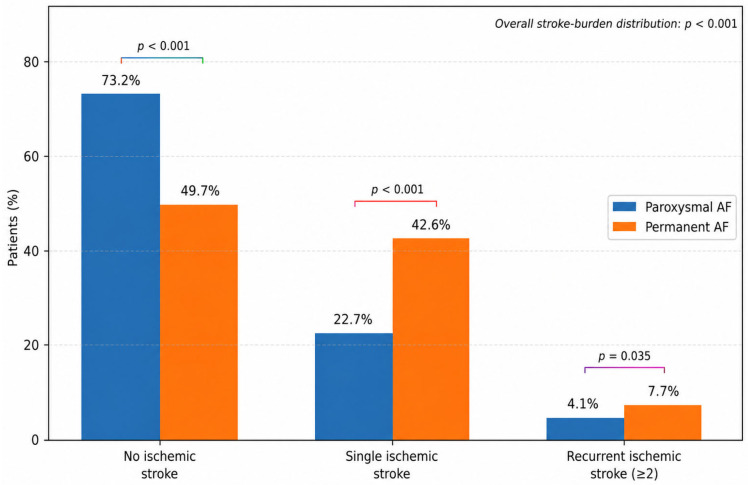
Stroke burden distribution according to AF phenotype.

Cerebrovascular outcomes according to AF phenotype are summarized in Table 3 and Figure 1. Permanent AF was associated with a significantly higher prevalence of any ischemic stroke. Any ischemic stroke was recorded in 325/646 patients with permanent AF (50.3%) compared with 79/295 patients with paroxysmal AF (26.8%; *p* < 0.001). Conversely, absence of ischemic stroke was more frequent in paroxysmal AF: 216/295 patients (73.2%) versus 321/646 patients (49.7%; *p* < 0.001).

Single ischemic stroke was also significantly more frequent in permanent AF. A single ischemic stroke event was recorded in 275/646 patients with permanent AF (42.6%) compared with 67/295 patients with paroxysmal AF (22.7%; *p* < 0.001). Recurrent ischemic stroke, defined as two or more events, was also more frequent in permanent AF—50/646 patients (7.7%) in permanent AF—versus 12/295 patients (4.1%) in paroxysmal AF (*p* = 0.035).

When recurrent ischemic stroke was evaluated only among patients who had already experienced at least one ischemic stroke, the proportions were nearly identical between AF phenotypes. Recurrent ischemic stroke among stroke-positive patients was present in 12/79 patients with paroxysmal AF (15.2%) and 50/325 patients with permanent AF (15.4%; *p* = 0.966). This suggests that permanent AF was strongly associated with the overall presence of cerebrovascular disease, but AF phenotype alone did not distinguish recurrent stroke burden once an ischemic stroke had already occurred.

The overall stroke burden distribution differed significantly between AF phenotypes (*p* < 0.001). The mean number of ischemic strokes per patient was 0.33 ± 0.65 in paroxysmal AF and 0.60 ± 0.70 in permanent AF (*p* < 0.001). The total number of recorded ischemic stroke events was 98 in the paroxysmal AF group and 390 in the permanent AF group.

### 3.5. TyG Index According to Ischemic Stroke Burden

**Figure 2:**
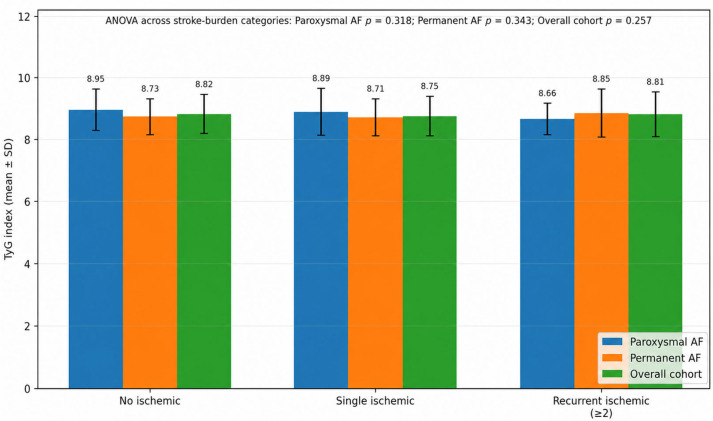
TyG index according to ischemic stroke burden.

Table 4 and Figure 2 presents triglyceride values, fasting plasma glucose, and TyG index according to ischemic stroke burden, stratified by AF phenotype and in the overall cohort. Stroke burden was categorized as no ischemic stroke, single ischemic stroke, or recurrent ischemic stroke.

In paroxysmal AF, triglyceride values, fasting plasma glucose, and TyG index did not differ significantly across stroke burden categories. The TyG index was 8.95 ± 0.67 in patients without ischemic stroke, 8.89 ± 0.76 in those with a single ischemic stroke, and 8.66 ± 0.51 in those with recurrent ischemic stroke (*p* = 0.318).

In permanent AF, no significant differences were observed across stroke burden categories for triglycerides, fasting plasma glucose, or TyG index. TyG index was 8.73 ± 0.58 in patients without ischemic stroke, 8.71 ± 0.60 in those with a single ischemic stroke, and 8.85 ± 0.78 in those with recurrent ischemic stroke (*p* = 0.343).

In the overall cohort, the TyG index did not differ significantly across stroke burden categories. Overall the TyG index values were 8.82 ± 0.63 in patients without ischemic stroke, 8.75 ± 0.64 in patients with a single ischemic stroke, and 8.81 ± 0.73 in those with recurrent ischemic stroke (*p* = 0.257). These findings indicate that the TyG index did not show a progressive increase with ischemic stroke burden in either AF phenotype or in the overall cohort.

### 3.6. Logistic Regression Analyses

**Figure 3:**
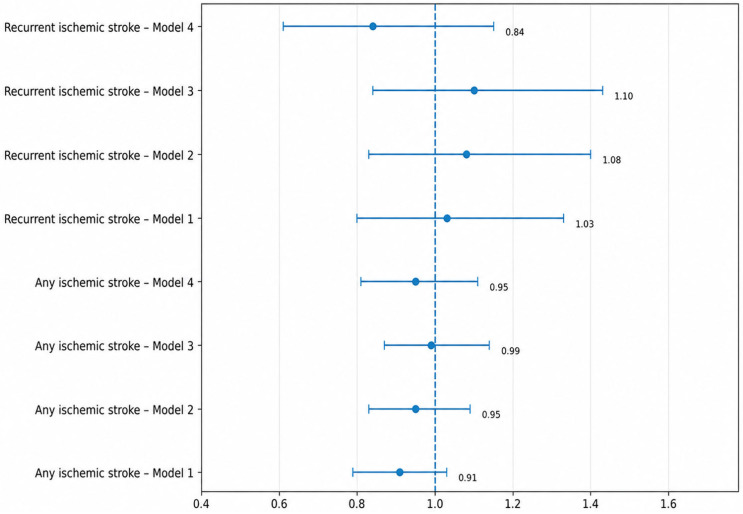
Logistic regression models for ischemic stroke outcomes.

Logistic regression models evaluating the association between the TyG index and ischemic stroke outcomes are presented in Table 5 and Figure 3. The TyG index was analyzed per 1-standard deviation increase, corresponding to 0.64 units in the analytical cohort.

For the outcome of any ischemic stroke, the TyG index was not significantly associated with the endpoint in the unadjusted model (OR 0.91, 95% CI 0.79–1.03; *p* = 0.138). This remained unchanged after adjustment for age and sex (OR 0.95, 95% CI 0.83–1.09; *p* = 0.458), after additional adjustment for AF phenotype (OR 0.99, 95% CI 0.87–1.14; *p* = 0.936), and in the clinically adjusted model including age, sex, AF phenotype, hypertension, diabetes mellitus, dyslipidemia, NYHA III–IV heart failure, oral anticoagulant therapy, and eGFR (OR 0.95, 95% CI 0.81–1.11; *p* = 0.513).

For recurrent ischemic stroke, the TyG index was also not significantly associated with the endpoint. In the unadjusted model, the OR per 1-standard deviation increase in TyG was 1.03 (95% CI 0.80–1.33; *p* = 0.820). The association remained non-significant after age and sex adjustment (OR 1.08, 95% CI 0.83–1.40; *p* = 0.581), after additional adjustment for AF phenotype (OR 1.10, 95% CI 0.84–1.43; *p* = 0.484), and in the clinically adjusted model (OR 0.84, 95% CI 0.61–1.15; *p* = 0.277).

CHA_2_DS_2_-VASc score was not included in the primary regression models because previous stroke or transient ischemic attack is a component of the score. Including the score in a model where ischemic stroke history is the dependent variable would introduce partial circularity. Instead, clinically relevant non-circular covariates were used for adjustment.

In the extended sensitivity model in Table 6, additional adjustment for recorded overweight/obesity status, hypertension grade, and number of antihypertensive drug classes did not materially change the association between the TyG index and ischemic stroke outcomes. The TyG index remained not independently associated with any ischemic stroke or recurrent ischemic stroke. Because recurrent ischemic stroke events were limited, the extended model for this endpoint should be interpreted as exploratory.

### 3.7. Summary of Results

Overall, permanent AF was associated with a markedly higher ischemic stroke burden than paroxysmal AF. However, the TyG index was higher in paroxysmal AF, the group with lower stroke burden, and was not significantly associated with either any ischemic stroke or recurrent ischemic stroke in descriptive, mean-based comparisons, ANOVA analyses, or adjusted logistic regression models. These results suggest a dissociation between the metabolic profile captured by the TyG index and cerebrovascular burden in this AF cohort. The excess stroke burden observed in permanent AF appears more likely related to age, AF phenotype, renal function, heart failure severity, anticoagulant exposure, and overall clinical risk accumulation than to TyG index alone.

## 4. Discussion

In this retrospective real-world cohort of patients with AF, permanent AF was associated with a substantially higher ischemic stroke burden than paroxysmal AF. Patients with permanent AF had a higher prevalence of any ischemic stroke, a higher proportion of single ischemic stroke events, and a higher mean number of ischemic strokes per patient. Recurrent ischemic stroke, defined as at least two recorded ischemic stroke events, was also more frequent in permanent AF in the full cohort. However, when the analysis was restricted to patients who had already experienced at least one ischemic stroke, the proportion with recurrent ischemic stroke was almost identical between AF phenotypes. The comparison between AF phenotypes also confirmed clinically relevant baseline imbalance. Patients with paroxysmal AF had a more adverse lipid metabolic profile, whereas patients with permanent AF were older and showed a more advanced clinical risk profile, including worse renal function and more frequent clinically significant heart failure. This imbalance supports cautious interpretation of crude between-group comparisons and justifies the use of sequentially adjusted regression models and the reviewer-requested extended sensitivity model incorporating available adiposity and hypertension treatment variables. Importantly, the neutral association between the TyG index and ischemic stroke outcomes persisted after this additional adjustment.

The second major finding is that the TyG index was not significantly associated with ischemic stroke burden in this dataset. The TyG index did not increase progressively across no-stroke, single stroke, and recurrent stroke categories, either in the overall cohort or after stratification by AF phenotype. In logistic regression, the TyG index was not associated with any ischemic stroke or recurrent ischemic stroke in unadjusted, age/sex-adjusted, AF phenotype-adjusted, or clinically adjusted models.

The third relevant finding is the dissociation between the metabolic profile captured by the TyG index and observed cerebrovascular burden. This pattern argues against a simple linear relationship between the TyG index and ischemic stroke burden in this heterogeneous AF population.

Our neutral findings should be interpreted in the context of a substantial body of literature reporting positive associations between the TyG index and cardiovascular or cerebrovascular outcomes. In general population cohorts, higher TyG index has been associated with increased risk of ischemic stroke and stroke subtypes, while meta-analytic data have also supported associations between the TyG index, stroke recurrence, and adverse outcomes after ischemic stroke [7,10,11,12,13,14,15,16,17,18,19,20,21]. In AF-specific literature, the TyG index has been linked with incident AF, AF recurrence after catheter ablation, and adverse cardiovascular or cerebrovascular outcomes in selected AF populations [8,22,23,24,25,26,27,28,29]. These data provide a biologically plausible rationale for evaluating the TyG index in AF cohorts, but they do not imply that TyG will have uniform prognostic value across all AF phenotypes and clinical settings.

The present study differs from many positive TyG studies in several important respects. Most prior studies evaluated incident stroke in general populations, prognosis after acute ischemic stroke, or selected cardiovascular subgroups. By contrast, the current analysis was based on a retrospective real-world cohort of patients with established AF and evaluated recorded ischemic stroke burden rather than prospectively adjudicated incident stroke. In stroke-positive patients, TyG reflected metabolic status during hospitalization rather than a pre-stroke exposure. In addition, medication exposure may have modified triglyceride and glucose values, while the absence of systematic stroke mechanism classification prevented testing whether TyG was more strongly related to atherosclerotic or small vessel mechanisms than to cardioembolic stroke. Therefore, this study evaluates cross-sectional association with accumulated cerebrovascular burden, not future stroke prediction.

Several features of the cohort may explain the lack of an independent TyG–stroke association. Patients with paroxysmal AF had a more adverse lipid metabolic profile and higher TyG index values, whereas patients with permanent AF had a higher stroke burden, older age, worse renal function, more advanced heart failure, and higher global clinical risk scores. This inverse distribution may have attenuated any crude association between the TyG index and cerebrovascular outcomes. In this context, AF phenotype and accumulated clinical risk appeared to be stronger determinants of stroke burden than the triglyceride–glucose metabolic axis alone.

The neutral result also supports the concept that the prognostic meaning of TyG index may be population-dependent and cardiometabolic-phenotype-dependent. TyG may be more informative in metabolically selected groups, in patients without established metabolic syndrome, in younger cohorts, in non-diabetic individuals, or when combined with anthropometric and inflammatory markers. In the present database, however, body weight, height, and waist circumference were unavailable, preventing calculation of TyG-BMI, TyG–waist circumference, conicity index, or TyG–conicity index. Therefore, the absence of an independent association with stroke burden should not be interpreted as excluding a metabolic contribution to cerebrovascular risk, but rather as indicating that TyG index alone had limited explanatory value in this heterogeneous AF cohort.

Overall, these findings refine rather than refute the TyG hypothesis. They suggest that the TyG index should not be used as a standalone marker of ischemic stroke burden in unselected AF populations, especially when stroke history, AF phenotype, renal function, heart failure severity, and anticoagulant exposure are major competing determinants of cerebrovascular risk. Future studies should evaluate TyG prospectively, with standardized timing of blood sampling, stroke mechanism classification, detailed anticoagulation data, and anthropometric or inflammatory measures.

Several mechanisms may explain why the TyG index was not significant in the present analysis. First, ischemic stroke in AF is mechanistically heterogeneous. Cardioembolism related to atrial remodeling and left atrial appendage thrombosis may coexist with large artery atherosclerosis, small vessel disease, renal dysfunction, heart failure, and treatment-related effects. The TyG index primarily captures a triglyceride–glucose metabolic axis and may not reflect atrial thrombus biology, anticoagulant adherence, time in therapeutic range, left atrial size, left atrial appendage function, or stroke mechanism classification.

Second, the distribution of the TyG index across AF phenotypes created an inverse clinical pattern. Paroxysmal AF patients had the more adverse lipid metabolic profile, while permanent AF patients had the higher stroke burden. This creates an internal dissociation that can weaken or neutralize a crude TyG–stroke association. From a statistical perspective, such a situation is compatible with confounding by AF phenotype and clinical risk accumulation; from a clinical perspective, it suggests that advanced AF substrate may outweigh the metabolic signal captured by TyG.

Third, the source database included a broad real-world AF population rather than a metabolically enriched subgroup. Positive TyG studies often show stronger signals in carefully defined populations, such as patients without diabetes, patients with small vessel occlusion, hypertensive patients, younger adults, post-stroke cohorts, or patients undergoing AF ablation [15,17,18,20,22,25]. In our dataset, patients differed substantially by AF phenotype, age, renal function, heart failure severity, diabetes, dyslipidemia, medication exposure, and prior cerebrovascular disease. This heterogeneity can dilute a biomarker association that might be present only in a narrower subgroup.

Fourth, the TyG index alone does not measure body fat distribution. This is important because the vascular consequences of insulin resistance depend not only on triglyceride and glucose concentrations, but also on visceral adiposity, ectopic fat, inflammatory tone, blood pressure, renal function, and the presence or absence of overt metabolic syndrome. Because waist circumference, body weight, and height were unavailable, conicity index, TyG-BMI, TyG–waist circumference, and TyG–conicity index could not be calculated. The negative result may therefore reflect the limited granularity of TyG alone rather than the absence of a metabolic contribution to stroke risk.

The present findings support the hypothesis that the prognostic meaning of TyG index is population-dependent and cardiometabolic-phenotype-dependent. This interpretation is supported by recent evidence showing effect modification by metabolic syndrome. Li et al. reported that the TyG index was associated with incident stroke overall and particularly among participants without metabolic syndrome, but not among participants with metabolic syndrome; the interaction between TyG and metabolic syndrome was statistically significant [30]. This suggests that TyG may operate differently depending on whether metabolic dysregulation is isolated, clustered, already clinically manifest, or accompanied by other dominant vascular risk factors.

Similarly, the U-shaped association reported between the TyG index and incident AF in the ARIC cohort suggests that both low and high TyG values may carry different biological meanings depending on population characteristics [24]. Low TyG in frail or chronically ill patients may reflect malnutrition, cachexia, inflammatory burden, advanced disease, or treatment effects, whereas high TyG may reflect insulin resistance, hypertriglyceridemia, and atherogenic metabolic dysfunction [31]. In older AF patients with heart failure and renal dysfunction, a lower TyG value is not necessarily metabolically protective. This may be relevant in the present cohort, where the permanent AF group had lower TyG values but a worse clinical risk profile and higher stroke burden [32].

Therefore, the neutral TyG result should be presented as a clinically meaningful finding. It indicates that in this database, TyG index does not independently explain ischemic stroke burden across paroxysmal and permanent AF. At the same time, it leaves open the possibility that TyG may be useful in selected AF subgroups: younger patients, patients without established metabolic syndrome, patients with preserved renal function, patients with predominant atherosclerotic rather than cardioembolic stroke mechanisms, non-diabetic individuals, or patients in whom TyG is combined with adiposity measures and inflammatory markers [33,34].

Clinically, these findings argue against using the TyG index as a standalone marker of ischemic stroke burden or recurrent stroke risk in heterogeneous AF populations. Established risk assessment in AF should remain centered on validated thromboembolic risk factors, anticoagulant eligibility, renal function, bleeding risk, heart failure severity, vascular disease, prior stroke/TIA, and longitudinal reassessment according to current AF guidelines [1,2].

However, the TyG index may still be useful as a low-cost marker of cardiometabolic risk, particularly when interpreted alongside diabetes status, lipid profile, obesity pattern, metabolic syndrome, renal function, inflammation, and AF phenotype. The negative result in the present cohort suggests that TyG should not be overgeneralized beyond the population in which it was validated. Rather than excluding TyG from future AF research, our data support a more refined approach: subgroup analyses, interaction testing, nonlinear modeling, and composite indices that integrate TyG with anthropometric and inflammatory markers.

### 4.1. Strengths

This study has several strengths. It includes a relatively large real-world AF cohort with two clinically distinct AF phenotypes. The database allows evaluation of ischemic stroke burden rather than only a binary stroke history. The analysis distinguishes between any ischemic stroke and recurrent ischemic stroke, which is clinically relevant because recurrence may reflect different mechanisms and prevention failures than first-event stroke. Finally, the TyG index was evaluated using descriptive comparisons, ANOVA across stroke burden categories, and sequentially adjusted logistic regression models, reducing the risk of overinterpreting crude metabolic differences.

### 4.2. Limitations

Several limitations should be acknowledged. First, the study is retrospective and observational; causality cannot be inferred. TyG was calculated from triglyceride and fasting glucose values measured during hospitalization; therefore, in stroke-positive patients, TyG was measured after the recorded stroke event or during the same clinical episode, not prospectively before stroke occurrence. The findings should therefore be interpreted as cross-sectional associations with recorded ischemic stroke burden rather than as evidence that TyG predicts future stroke risk.

Second, time in therapeutic range for vitamin K antagonist users, detailed anticoagulant adherence, and anticoagulant dose appropriateness were not available. Although ischemic stroke was clinically documented and CT-confirmed, etiological stroke mechanism classification was not systematically recorded. Therefore, we could not evaluate whether TyG was differentially associated with cardioembolic, large artery, small vessel, mixed, or undetermined stroke mechanisms.

Third, TyG values may have been influenced by statins, antidiabetic therapy, nutritional status, or acute illness, because TyG reflects treated metabolic status at admission. Fourth, continuous BMI, waist circumference, and target blood pressure achievement were not systematically recorded. Although recorded overweight/obesity categories, hypertension grade, and antihypertensive treatment variables were incorporated into additional analyses, residual confounding related to adiposity distribution, body size phenotype, and quality of blood pressure control cannot be excluded.

Fifth, recurrent ischemic stroke events were relatively limited in number, especially in the paroxysmal AF group, restricting model complexity and limiting the stability of subgroup analyses. Residual confounding may also persist because structural cardiac variables, left atrial appendage parameters, inflammatory markers, nutritional status, and the severity of atherosclerotic disease were not systematically available. Finally, CHA_2_DS_2_-VASc score was not included in the primary regression models because previous stroke or transient ischemic attack is a component of the score. Although clinically relevant, including this score in models where stroke history is the dependent variable would introduce partial circularity.

### 4.3. Future Directions

Future studies should evaluate the TyG index prospectively in AF cohorts with standardized timing of blood sampling, detailed anticoagulation data, stroke mechanism classification, left atrial and left atrial appendage imaging, inflammatory biomarkers, and anthropometric measures. Particular attention should be given to interaction analyses by diabetes status, metabolic syndrome, obesity phenotype, renal function, AF phenotype, and stroke mechanism. Such studies may clarify whether TyG index is most useful as a general cardiometabolic risk marker, as an adjunct to existing thromboembolic risk scores, or only as a subgroup-specific biomarker.

## 5. Conclusions

In this retrospective cohort of patients with AF derived from a real-world clinical database, permanent AF was associated with a significantly higher burden of ischemic stroke than paroxysmal AF. However, TyG index was not independently associated with any ischemic stroke or recurrent ischemic stroke. Although patients with paroxysmal AF showed higher triglyceride values and higher TyG index values, this metabolic profile did not translate into a higher TyG-related cerebrovascular burden.

These findings demonstrate that, in this specific dataset, the TyG index alone does not explain ischemic stroke burden. The result contrasts with several positive studies in general populations, post-stroke cohorts, and selected AF cohorts, and should be interpreted as evidence that TyG-related risk is likely dependent on population structure and cardiometabolic phenotype. In heterogeneous AF patients, cerebrovascular complications appear to be driven more strongly by AF phenotype, age, renal function, heart failure severity, anticoagulant exposure, and established thromboembolic risk factors than by triglyceride–glucose-derived metabolic risk alone.

Future prospective studies incorporating anthropometric indices, conicity index, TyG-BMI, TyG–waist circumference, inflammatory biomarkers, detailed anticoagulation quality, left atrial structural markers, and stroke mechanism classification are needed to determine whether more complex metabolic–clinical models can improve cerebrovascular risk stratification in AF.

## Figures and Tables

**Table 1:** Baseline clinical characteristics according to atrial fibrillation phenotype.

Variable	Paroxysmal AF, n = 295	Permanent AF, n = 646	*p*-Value
Age, years	69.7 ± 10.8	73.6 ± 10.0	<0.001
Female sex	146/295 (49.5)	348/646 (53.9)	0.212
Urban residence	180/295 (61.0)	299/646 (46.3)	<0.001
Multiple hospitalizations	64/295 (21.7)	135/646 (20.9)	0.781
Hospital stay, days	8.1 ± 4.2	8.3 ± 4.4	0.422
CHA2DS2-VASc score	4.5 ± 1.8	5.2 ± 1.8	<0.001
HAS-BLED score	3.9 ± 1.2	4.3 ± 1.2	<0.001
ATRIA score	2.9 ± 2.3	3.5 ± 2.3	<0.001
eGFR, mL/min/1.73 m2	57.7 ± 22.4	53.2 ± 21.9	0.004
Hypertension	264/295 (89.5)	548/646 (84.8)	0.054
Diabetes mellitus	120/295 (40.7)	185/646 (28.6)	<0.001
Dyslipidemia	228/295 (77.3)	409/646 (63.3)	<0.001
NYHA III–IV heart failure	79/295 (26.8)	296/646 (45.8)	<0.001
Coronary artery disease	195/295 (66.1)	394/646 (61.0)	0.133
Carotid stenosis	12/295 (4.1)	28/646 (4.3)	0.851
Oral anticoagulant therapy	228/295 (77.3)	570/646 (88.2)	<0.001
Statin therapy	203/295 (68.8)	362/646 (56.0)	<0.001
Aspirin therapy	68/295 (23.1)	60/646 (9.3)	<0.001
Clopidogrel therapy	57/295 (19.3)	61/646 (9.4)	<0.001

Values are presented as mean ± standard deviation or n/N (%).

**Table 2:** Biological and metabolic profile according to atrial fibrillation phenotype.

Variable	Paroxysmal AF, n = 295	Permanent AF, n = 646	*p*-Value
Total cholesterol, mg/dL	160.4 ± 52.2	149.7 ± 48.4	0.003
HDL-C, mg/dL	41.8 ± 15.7	43.6 ± 17.6	0.110
LDL-C, mg/dL	99.1 ± 43.5	91.0 ± 38.8	0.007
Triglycerides, mg/dL	127.5 ± 77.9	105.8 ± 58.7	<0.001
Fasting plasma glucose, mg/dL	146.2 ± 71.9	139.3 ± 64.8	0.162
TyG index	8.93 ± 0.69	8.73 ± 0.61	<0.001

Values are presented as mean ± standard deviation.

**Table 3:** Cerebrovascular burden according to atrial fibrillation phenotype.

Variable	Paroxysmal AF, n = 295	Permanent AF, n = 646	*p*-Value
No ischemic stroke	216/295 (73.2)	321/646 (49.7)	<0.001
Any ischemic stroke	79/295 (26.8)	325/646 (50.3)	<0.001
Single ischemic stroke	67/295 (22.7)	275/646 (42.6)	<0.001
Recurrent ischemic stroke, ≥2 events	12/295 (4.1)	50/646 (7.7)	0.035
Recurrent ischemic stroke among stroke-positive patients	12/79 (15.2)	50/325 (15.4)	0.966
Stroke burden distribution: 0/1/≥2 events	216/67/12	321/275/50	<0.001
Number of ischemic strokes per patient	0.33 ± 0.65	0.60 ± 0.70	<0.001
Total recorded ischemic stroke events	98	390	—

Values are presented as mean ± standard deviation or n/N (%). Recurrent ischemic stroke was defined as ≥2 recorded ischemic stroke events.

**Table 4:** Comparison of metabolic parameters according to ischemic stroke burden.

AF Phenotype/Variable	No Ischemic Stroke	Single Ischemic Stroke	Recurrent Ischemic Stroke, ≥2 Events	*p*-Value
Paroxysmal AF	n = 216	n = 67	n = 12	
Triglycerides, mg/dL	131.7 ± 77.3	121.0 ± 84.1	88.2 ± 28.2	0.125
Fasting plasma glucose, mg/dL	142.9 ± 65.3	155.9 ± 90.0	149.8 ± 73.1	0.432
TyG index	8.95 ± 0.67	8.89 ± 0.76	8.66 ± 0.51	0.318
Permanent AF	n = 321	n = 275	n = 50	
Triglycerides, mg/dL	105.3 ± 46.2	105.3 ± 68.6	111.9 ± 71.3	0.752
Fasting plasma glucose, mg/dL	136.7 ± 62.1	139.2 ± 63.4	156.6 ± 85.3	0.130
TyG index	8.73 ± 0.58	8.71 ± 0.60	8.85 ± 0.78	0.343
Overall cohort	n = 537	n = 342	n = 62	
Triglycerides, mg/dL	115.9 ± 62.0	108.4 ± 72.0	107.3 ± 65.7	0.206
Fasting plasma glucose, mg/dL	139.2 ± 63.4	142.4 ± 69.6	155.3 ± 82.6	0.193
TyG index	8.82 ± 0.63	8.75 ± 0.64	8.81 ± 0.73	0.257

Values are presented as mean ± standard deviation. The *p*-value refers to comparison across the three stroke burden categories using one-way analysis of variance.

**Table 5:** Logistic regression models evaluating the association between TyG index and ischemic stroke outcomes.

Outcome/Model	n	Events	TyG OR per 1-SD Increase	*p*-Value
Any ischemic stroke				
Model 1: unadjusted	941	404	0.91 (0.79–1.03)	0.138
Model 2: age + sex-adjusted	941	404	0.95 (0.83–1.09)	0.458
Model 3: + AF phenotype	941	404	0.99 (0.87–1.14)	0.936
Model 4: clinical adjustment	941	404	0.95 (0.81–1.11)	0.513
Recurrent ischemic stroke, ≥2 events				
Model 1: unadjusted	941	62	1.03 (0.80–1.33)	0.820
Model 2: age + sex-adjusted	941	62	1.08 (0.83–1.40)	0.581
Model 3: + AF phenotype	941	62	1.10 (0.84–1.43)	0.484
Model 4: clinical adjustment	941	62	0.84 (0.61–1.15)	0.277

Odds ratios are reported for a 1-standard deviation increase in TyG index. Model 4 was adjusted for age, sex, AF phenotype, hypertension, diabetes mellitus, dyslipidemia, NYHA III–IV heart failure, oral anticoagulant therapy, and eGFR.

**Table 6:** Extended sensitivity regression model including available adiposity and hypertension treatment variables.

Outcome/Model	n	Events	TyG OR per 1-SD Increase	*p*-Value
Any ischemic stroke, Model 5	941	404	0.94 (0.80–1.11)	0.464
Recurrent ischemic stroke, ≥2 events, Model 5	941	62	0.84 (0.61–1.16)	0.293

Odds ratios are reported for a 1-standard deviation increase in TyG index. Model 5 was adjusted for age, sex, AF phenotype, diabetes mellitus, dyslipidemia, NYHA III–IV heart failure, oral anticoagulant therapy, eGFR, recorded overweight/obesity status, hypertension grade I–III, and number of antihypertensive drug classes. Continuous BMI, waist circumference, and target blood pressure achievement were not available and therefore could not be directly included in the model.

## Data Availability

The original contributions presented in this study are included in the article material. Further inquiries can be directed to the corresponding author.

## Supplementary Materials

The following supporting information can be downloaded at: https://www.mdpi.com/article/10.3390/metabo16070482/s1.

## Abbreviations

The following abbreviations are used in this manuscript:

AF	atrial fibrillation
CI	confidence interval
eGFR	estimated glomerular filtration rate
HDL-C	high-density lipoprotein cholesterol
LDL-C	low-density lipoprotein cholesterol
NYHA	New York Heart Association
OR	odds ratio
TyG	triglyceride–glucose
