# Supplementary material for: Triglyceride–Glucose Index and Ischemic Stroke Burden in Permanent Versus Paroxysmal Atrial Fibrillation: A Real-World Retrospective Cohort Study
Source: Metabolites. 2026 Jul 9;16(7):482. doi: 10.3390/metabo16070482 (PMC13413521; doi:10.3390/metabo16070482)
Supplement: Supplementary file 1 [file metabolites-16-00482-s001.zip › Suplementary Table S1.pdf]

Supplementary Table S1

Table S1. Cohort accountability and manuscript-aligned TyG cohort construction

Step	Overall	Paroxysmal AF	Permanent AF	Comment
Initial AF-related records in the raw database	1,456	501	955	Before patient-level de-duplication
Unique patients after de-duplication	1,031	310	721	
TyG-eligible analytical cohort	941	295	646	Valid triglyceride/glucose data and aligned with manuscript analytical cohort
Excluded from TyG-specific analyses	90	15	75	Missing/non-valid triglyceride and/or glucose data, including one paroxysmal AF patient with triglycerides coded as unavailable

Note. AF = atrial fibrillation; TyG = triglyceride–glucose index. Patient-level de-duplication used normalized AF group and patient number to avoid treating repeated admissions as independent patients.

Table S1.1 Comparison of patients included in versus excluded from TyG-specific analyses

Variable	TyG-eligible cohort, n = 941	Excluded from TyG analysis, n = 90	p-value
Age, years	72.4 ± 10.4	76.0 ± 10.3	0.002
Female sex	495/941 (52.6)	46/90 (51.1)	0.786
Paroxysmal AF	295/941 (31.3)	15/90 (16.7)	0.004
Permanent AF	646/941 (68.7)	75/90 (83.3)	0.004
Urban residence	480/941 (51.0)	43/90 (47.8)	0.558
Multiple hospitalizations	190/941 (20.2)	7/90 (7.8)	0.004
Hospital stay, days	8.2 ± 4.3	6.3 ± 5.0	<0.001
CHA_2DS_2-VASc score	5.0 ± 1.9	5.6 ± 2.0	0.011
HAS-BLED score	4.1 ± 1.2	4.4 ± 1.4	0.062
ATRIA score	3.3 ± 2.3	3.8 ± 2.7	0.127
eGFR, mL/min/1.73 m^2	54.6 ± 22.1	46.3 ± 26.7	0.005
Hypertension	801/941 (85.1)	66/90 (73.3)	0.003
Diabetes mellitus	284/941 (30.2)	29/90 (32.2)	0.687
Dyslipidemia	620/941 (65.9)	47/90 (52.2)	0.010
NYHA III–IV heart failure	274/941 (29.1)	37/90 (41.1)	0.018
Coronary artery disease	563/940 (59.9)	56/90 (62.2)	0.667
Carotid stenosis	31/941 (3.3)	4/90 (4.4)	0.538
Oral anticoagulant therapy	718/940 (76.4)	45/90 (50.0)	<0.001
Statin therapy	524/941 (55.7)	24/90 (26.7)	<0.001
Aspirin therapy	114/941 (12.1)	4/90 (4.4)	0.029
Clopidogrel therapy	99/941 (10.5)	7/90 (7.8)	0.413
Any ischemic stroke	404/941 (42.9)	56/90 (62.2)	<0.001
Recurrent ischemic stroke, ≥2 events	62/941 (6.6)	8/90 (8.9)	0.407

Note. Values are mean ± standard deviation or n/N (%). p-values were calculated using Welch's t-test for continuous variables and chi-square or Fisher's exact test for categorical variables, as appropriate. This table is intended to address potential selection bias related to missing or non-valid triglyceride/glucose data.

Table S1.2 Reasons for exclusion from TyG-specific analyses

Reason for exclusion from TyG analysis	Overall	Paroxysmal AF	Permanent AF
Missing/non-valid triglycerides	73	13	60
Missing/non-valid glucose	10	2	8
Missing/non-valid triglycerides and glucose	7	0	7
