# Supplementary material for: Triglyceride–Glucose Index and Ischemic Stroke Burden in Permanent Versus Paroxysmal Atrial Fibrillation: A Real-World Retrospective Cohort Study
Source: Metabolites. 2026 Jul 9;16(7):482. doi: 10.3390/metabo16070482 (PMC13413521; doi:10.3390/metabo16070482)
Supplement: Supplementary file 1 [file metabolites-16-00482-s001.zip › Suplementary Table S2.pdf]

Table S2. Regression diagnostics for logistic regression models

Outcome	Model	n	Events	Predictors	EPV	Converged	Maximum VIF	Hosmer–Lemeshow p-value	C-statistic/AUC
Any ischemic stroke	Model 1: unadjusted	941	404	1	404.0	Yes	—	0.185	0.535
Any ischemic stroke	Model 2: age + sex adjusted	941	404	3	134.7	Yes	1.06	0.285	0.604
Any ischemic stroke	Model 3: + AF phenotype	941	404	4	101.0	Yes	1.09	0.044	0.647
Any ischemic stroke	Model 4: clinical adjustment	940	404	10	40.4	Yes	1.40	0.517	0.660
Recurrent ischemic stroke, ≥2 events	Model 1: unadjusted	941	62	1	62.0	Yes	—	0.277	0.494
Recurrent ischemic stroke, ≥2 events	Model 2: age + sex adjusted	941	62	3	20.7	Yes	1.06	0.979	0.624
Recurrent ischemic stroke, ≥2 events	Model 3: + AF phenotype	941	62	4	15.5	Yes	1.09	0.204	0.644
Recurrent ischemic stroke, ≥2 events	Model 4: clinical adjustment	940	62	10	6.2	Yes	1.40	0.436	0.736

Note. EPV = events per predictor; VIF = variance inflation factor; AUC = area under the receiver-operating-characteristic curve. Predictors are counted excluding the intercept. Model 4 included TyG index, age, sex, AF phenotype, hypertension, diabetes mellitus, dyslipidemia, NYHA III–IV heart failure, oral anticoagulant therapy, and eGFR.

Table S2.1 Recalculated TyG associations from logistic regression models

Outcome	Model	n	Events	TyG OR per 1-SD increase (95% CI)	p-value
Any ischemic stroke	Model 1: unadjusted	941	404	0.91 (0.80–1.04)	0.152
Any ischemic stroke	Model 2: age + sex adjusted	941	404	0.95 (0.83–1.09)	0.496
Any ischemic stroke	Model 3: + AF phenotype	941	404	1.00 (0.87–1.15)	0.994
Any ischemic stroke	Model 4: clinical adjustment	940	404	0.93 (0.79–1.10)	0.390
Recurrent ischemic stroke, ≥2 events	Model 1: unadjusted	941	62	1.03 (0.80–1.33)	0.808
Recurrent ischemic stroke, ≥2 events	Model 2: age + sex adjusted	941	62	1.08 (0.83–1.41)	0.569
Recurrent ischemic stroke, ≥2 events	Model 3: + AF phenotype	941	62	1.10 (0.85–1.44)	0.471
Recurrent ischemic stroke, ≥2 events	Model 4: clinical adjustment	940	62	0.83 (0.60–1.14)	0.248

Note. Odds ratios are reported per 1-standard-deviation increase in TyG index. These values are included only to support internal verification; the main manuscript table should be kept consistent with the final chosen cohort and covariate definitions.
