# Supplementary material for: Triglyceride–Glucose Index and Ischemic Stroke Burden in Permanent Versus Paroxysmal Atrial Fibrillation: A Real-World Retrospective Cohort Study
Source: Metabolites. 2026 Jul 9;16(7):482. doi: 10.3390/metabo16070482 (PMC13413521; doi:10.3390/metabo16070482)
Supplement: Supplementary file 1 [file metabolites-16-00482-s001.zip › Suplementary Table S3.pdf]

Supplementary Table S3

Table S3. Adiposity, hypertension grade, and antihypertensive-treatment profile according to atrial fibrillation phenotype

Variable	Paroxysmal AF, n = 295	Permanent AF, n = 646	p-value
Overweight	15/295 (5.1)	52/646 (8.0)	0.133
Any obesity	85/295 (28.8)	173/646 (26.8)	0.569
Overweight or obesity	100/295 (33.9)	225/646 (34.8)	0.838
Obesity class I	46/295 (15.6)	93/646 (14.4)	0.703
Obesity class II	26/295 (8.8)	39/646 (6.0)	0.156
Obesity class III	13/295 (4.4)	41/646 (6.3)	0.300
Hypertension grade I	18/295 (6.1)	36/646 (5.6)	0.863
Hypertension grade II	107/295 (36.3)	256/646 (39.6)	0.363
Hypertension grade III	128/295 (43.4)	257/646 (39.8)	0.331
Beta-blocker therapy	230/295 (78.0)	496/646 (76.8)	0.750
Calcium-channel blocker therapy	73/295 (24.7)	136/646 (21.1)	0.238
Diuretic therapy	171/295 (58.0)	464/646 (71.8)	<0.001
ACE inhibitor therapy	106/295 (35.9)	249/646 (38.5)	0.487
Angiotensin receptor blocker therapy	40/295 (13.6)	51/646 (7.9)	0.009
Any antihypertensive treatment	265/295 (89.8)	575/646 (89.0)	0.792
Number of antihypertensive drug classes	2.1 ± 1.1	2.2 ± 1.1	0.444

Note. Values are presented as mean ± standard deviation or n/N (%), as appropriate. ACE = angiotensin-converting enzyme; AF = atrial fibrillation; BMI = body mass index. BMI and waist circumference were not available as continuous measured variables in the source database. Target blood pressure achievement could not be assessed because standardized blood pressure target data were not systematically recorded.
